# Valgus Osteotomy with DHS Fixation in the Management of Malunited Intertrochanteric Fractures in a Rural Population

**DOI:** 10.5704/MOJ.2011.015

**Published:** 2020-11

**Authors:** Y Subash

**Affiliations:** Department of Orthopaedics, Saveetha University Saveetha Medical College and Hospital, Kanchipuram, India

**Keywords:** intertrochanteric fractures, malunion, valgus osteotomy, DHS

## Abstract

**Introduction:**

Malunited intertrochanteric fractures are frequently seen in rural populations as they tend to go in for native treatment with traditional bone setters. The resulting Coxa vara is associated with shortening, abductor weakness, limp and decreased range of movement of the affected hip. The aim of this study was to evaluate the role of Valgus osteotomy with Dynamic hip screw (DHS) fixation in the management of these fractures and to evaluate the functional outcome using the Harris hip score.

**Materials and Methods:**

Fifteen patients with malunited intertrochanteric fractures who presented between January 2011 to January 2013 were managed by Valgus osteotomy with DHS fixation and were followed-up for a minimum period of three years.

**Results:**

There was a male preponderance seen in our study with the right hip being more commonly affected. The most common mode of injury was slip and fall followed by road traffic accidents. The duration of native treatment ranged from seven to 12 weeks and the time of presentation to the hospital ranged from four to nine months following injury. Pre-operative mean neck shaft angle was 94.73° while it was 134.6° post-operatively. The mean pre-operative Harris hip score was 72.33 and it was 91 at follow-up. All patients were happy with the procedure and the functional outcome.

**Conclusion:**

Valgus osteotomy with DHS fixation is an effective procedure in the management of malunited intertrochanteric fractures. It corrects the limb length discrepancy, restores the decreased neck shaft angle, improves range of movement, restores the integrity of the abductor mechanism of the hip and gives good functional results.

## Introduction

Intertrochanteric fractures of the hip are common fractures seen in an elderly age group due to poor bone stock while in younger individuals they often result from high velocity trauma such as fall from a height and road traffic accidents^1,2^. Various surgical options are available in the management of these fractures. In developing countries patients do tend to present late for treatment due to various factors. Our hospital is located in a rural area where most of the population belong to a lower socio economic group and a bulk of them are manual laborers who depend on daily wages for their sustenance. So, factors such as lack of affordability and also traditional belief in native bone setters make them go in for native treatment with crude native splints extending from the groin to the toes. The period of native treatment often lasts for around six to eight weeks with the splints being changed at intervals of two weeks. There is often a history of massage or manipulation before the splints are applied leading to an increased risk of development of myositis ossificans. Since the intertrochanteric region is composed primarily of cancellous bone these fractures tend to unite well albeit in a nonanatomic manner leading to a malunion. The resulting Coxa vara is associated with problems such as shortening, external rotation deformity with limitation of abduction and internal rotation, limp, and issues such as hip and back pain due to mechanical factors^3-5^.

Due to decrease in the range of movement these patients find it difficult to squat and sit cross legged which are necessary for them for purposes of defecation and for sitting on the ground for partaking meals respectively and hence it becomes a disability for them which brings them to the hospital for treatment at a later stage.

The ideal treatment for failed and malunited intertrochanteric fractures as mentioned in literature has been a total hip arthroplasty but it would not be ideal in this situation because it is an expensive procedure and the lack of affordability and the avoidance of squatting and sitting cross legged following arthroplasty which would not be feasible for the patients who present from a rural population. A review of literature as well as articles from various authors have not described any other procedure in the management of these malunited fractures. Hence there is a need for a procedure which will restore limb length discrepancy, bring back the reduced neck shaft angle to as near normal as possible, improve the range of movement of the hip facilitating squatting and sitting cross legged and restore the integrity of the abductor mechanism and which gives good reproducible results and is also cost effective to the patient. In this scenario, Valgus osteotomy with DHS fixation would be ideally suited and would address all the above mentioned issues.

Valgus osteotomy is an established procedure which has been primarily used in the management of femoral neck fractures especially in the younger population where there arises a need to salvage the femoral head. It has not been described traditionally in the management of these malunited intertrochanteric fractures. The aim of this study was to evaluate the role of this procedure in the management of malunited intertrochanteric fractures and to analyse the functional results using the Harris hip score.

## Materials and Methods

This was a prospective study of 15 patients with malunited intertrochanteric fractures managed by Valgus osteotomy with DHS fixation between January 2011 to January 2013. This study was approved by the ethical committee of our institution All patients with malunited intertrochanteric fractures who were willing for the procedure and for regular follow-up were included in the study while patients not willing for follow-up and patients with active hip or systemic infection were excluded. On presentation to the hospital a thorough physical examination was done and findings such as amount of shortening, range of motion of the affected hip and the pre-operative Harris hip score were assessed and documented in the case records. The patients were then evaluated radiologically by taking radiographs of the pelvis with both hips anteroposterior view and affected hip lateral view and the neck shaft angle was measured and documented. Extensive pre-operative planning was not done because the aim was not to take a wedge of bone but only to perform a sliding osteomy at the subtrochanteric level in order to minimise shortening. All patients were then worked up for the surgical procedure.

**Fig. 1:**
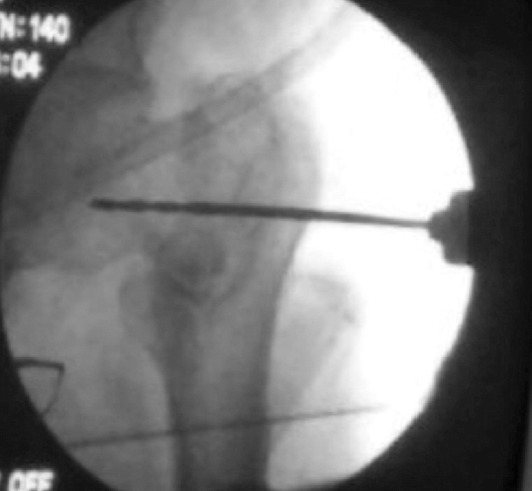
Entry point made using a 3.2mm drill bit.

**Fig. 2:**
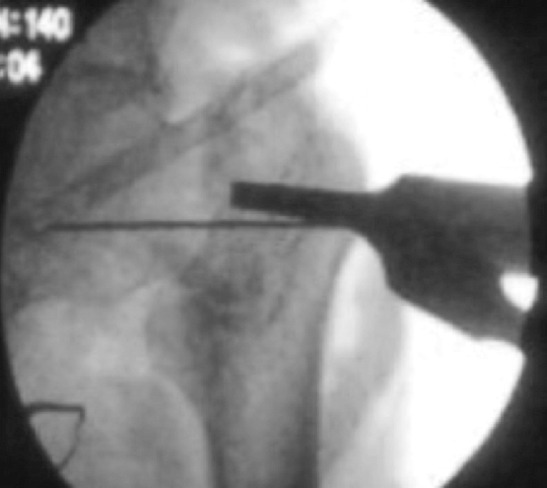
Guide wire passed parallel and inferior to the femoral neck under fluoroscopic guidance.

**Fig. 3:**
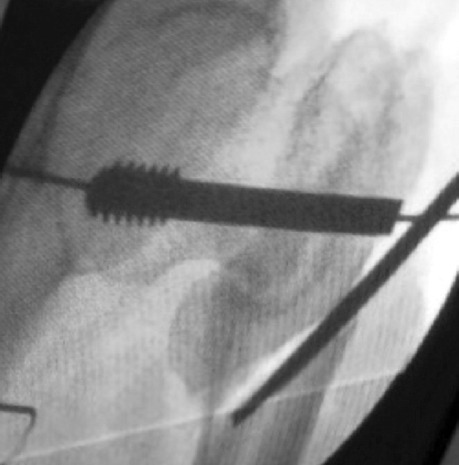
Richards screw inserted and osteotomy performed at the subtrochanteric level.

**Fig. 4:**
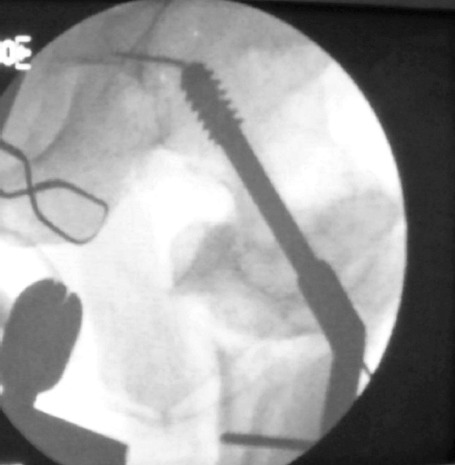
DHS barrel and plate applied with the restoration of the neck shaft angle to an anatomic position.

The patients were then taken up for surgery after obtaining anaesthetic fitness and proper informed consent for the procedure. The surgery was performed under spinal anaesthesia with the patient in the supine position on the fracture table under fluoroscopic guidance. The proximal femur was exposed via a standard lateral approach and after making an entry point using a 3.2mm drill bit, a guide wire was passed in the centre of the femoral neck and position was confirmed in anteroposterior and lateral views (Fig. 1 and 2). Reaming was then performed with a triple reamer and an appropriate size Richards screw was then applied. No effort was made to osteotomize the fracture site. An oblique osteotomy was then performed using an oscillating saw at a level just distal to the level of the lesser trochanter without taking a wedge of bone in order to minimise post-operative shortening (Fig. 3 and 4). Care was taken not to perform the osteotomy too distal to the subtrochanteric region in order to avoid problems with union at the osteotomy site. After performing the osteotomy, the external rotation deformity was corrected, a 5 holed DHS plate with a short barrel was then applied, the limb was taken into abduction and traction was reduced in order to reduce the shaft to the proximal fragment and the plate was fixed with five 4.5mm cortical screws. A DHS with a standard 135° angle was used in all cases. The limb was then brought back to a neutral position where the restoration of the limb length as well as the neck shaft angle could be appreciated. After ensuring heamostasis and placing a drain insitu the wound was closed in layers and sterile dressing was applied.

The patients were made to sit up in bed the same evening of surgery and the knee and ankle were actively mobilised. They were made to stand and walk on the first or second post-operative day with non-weight bearing walking with walking frame support subjective to pain tolerance and patient compliance. Drain removal was done on the third day and intravenous antibiotics were given for three days postoperatively. Wound inspections were done on the third and sixth post-operative day followed by suture removal on the 10th post-operative day. Limb length measurement, range of movement of the hip and the neck shaft angle on the postoperative radiographs were noted and documented. Patients were then started on quadriceps and hip abductor strengthening exercises and on discharge were asked to continue strict non-weight bearing until advised and were asked to review for follow-up at one, three, six months and at yearly intervals for three years. Radiological evaluations were done by taking serial radiographs at follow-up and functional evaluation was done using the Harris hip score and documented. Trendelenberg's test was performed in all patients to assess the integrity of the abductor mechanism. The data collected was analysed using IBM [SPSS Version 22.0. Armonk, NY: IBM Corp]. Chi square test was used in the comparison of categorical variables. A P value of less than 0.05 was considered to be statistically significant.

## Results

**Fig. 5:**
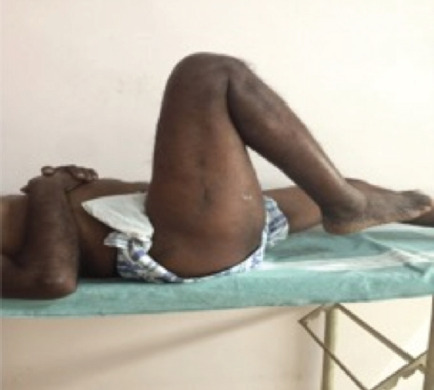
Range of motion-Hip flexion.

**Fig. 6:**
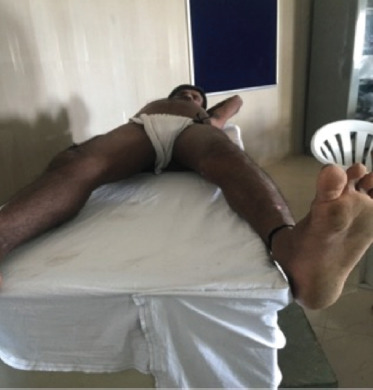
Range of motion-Hip Abduction.

**Fig. 7:**
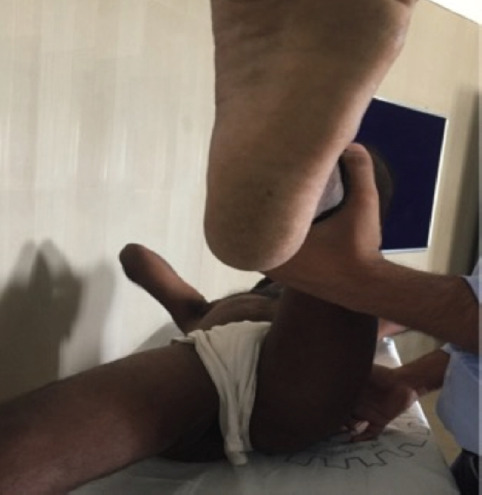
Range of motion-Hip Internal rotation.

There were 15 patients in our study with 13 males and two females. The right hip was more commonly affected as seen in nine patients. The age of the patients ranged from 49 to 70 years with the mean age being 61.53 years. The most common mode of injury was slip and fall followed by road traffic accidents and fall from a height. The time from injury to presentation to the hospital ranged from four to nine months with the mean being 6.2 months. The duration of native treatment ranged from seven to 12 weeks with the mean being 9.33 weeks. The average neck shaft angle pre-operatively was 94.73° ranging from 90° to 102° while it was 134.6° in the post-operative period ranging from 130° to 140°. The average pre-operative shortening was 2.66cm ranging from two to 3.1cm while three patients had minimal shortening in the post-operative period of about 0.2 to 0.5cm which was well tolerated by the patients. The average preoperative Harris hip score was 72.33 ranging from 61 to 80 while it was 91 in the post-operative period ranging from 80 to 97 with a gain of 18.67 points. The average duration of surgery was 98.5 minutes and the average blood loss was 209.4 milliliters. The average time to union of the osteotomy site was 12.7 weeks ranging from 11 to 15 weeks (Table I). All patients had limitation of abduction and internal rotation pre-operatively which improved significantly in the postoperative period with the mean flexion of more than 90°, abduction-40°, adduction-25°, internal rotation-45°, external rotation-30° (Fig. 5,6 and 7 and Table II). There were no complications such as superficial or deep infection, screw backout, joint penetration, loss of fixation or correction or non-union at the osteotomy site encountered in our study. None of the patients were lost to follow-up. All patients were happy with the procedure and the functional outcome.

**Table I:** Patient demographics and data

			Neck shaft angle (degrees)		Shortening (cm)		Harris hip score		Osteotomy site union (weeks)
Serial number	Age/ sex	Mode of injury	Pre-op	Post-op	Pre-op	Post-op	Pre-op	Post-op	
1	54/M	RTA	90	135	3	0.5	48	76	13
2	56/M	RTA	91	134	3	0.4	47	79	11
3	49/F	FFH	95	135	2.7	0	50	84	11
4	64/M	SAF	100	140	2	0	51	79	12
5	70/M	SAF	102	136	2.1	0	47	82	13
6	65/M	SAF	98	134	2.8	0	48	84	13
7	59/F	RTA	95	135	3	0.2	55	77	15
8	62/M	SAF	96	134	2.6	0	49	81	14
9	64/M	SAF	92	134	2.6	0	48	79	11
10	70/M	SAF	90	135	3.1	0	53	75	12
11	63/M	RTA	98	137	2.8	0	49	80	15
12	59/M	FFH	94	134	2.5	0	50	79	15
13	61/M	SAF	96	135	2.7	0	47	84	11
14	69/M	SAF	90	135	3	0	54	83	12
15	69/M	RTA	94	130	2	0	50	80	13

*RTA-Road traffic accident. SAF-Slip and fall. FFH-Fall from a height

**Table II:** The pre and post-operative range of movement

S.No	Pre-operative range of movement					Post-operative range of movement				
	Flex	Abd	Add	IR	ER	Flex	Abd	Add	IR	ER
1	90	12	15	5	10	120	40	25	40	25
2	85	10	20	6	15	110	42	20	42	30
3	87	14	20	10	20	100	45	21	39	35
4	80	9	25	11	15	120	39	20	44	45
5	75	11	20	8	14	110	40	25	40	40
6	70	10	15	6	21	100	38	20	41	35
7	86	12	21	9	20	120	41	20	42	40
8	70	13	15	10	15	110	42	24	44	41
9	85	10	25	10	18	100	37	21	41	45
10	80	9	20	12	21	110	40	20	43	40
11	90	15	15	11	15	120	42	20	40	38
12	80	11	20	13	20	100	45	25	39	40
13	85	9	15	9	21	110	44	21	38	45
14	75	10	18	12	20	100	40	25	42	42
15	70	12	24	11	18	100	42	20	45	45

* Flex-Flexion. Abd-Abduction. Add-Adduction. IR-Internal rotation. ER-External rotation

## Discussion

The management of fresh intertrochanteric fractures are quite straight forward with various treatment options and implants available for the same. In case of failed management of the fractures, the patients present with a malunion with coxa vara which results in mechanical block with restriction of abduction and internal rotation as well as shortening of the affected limb which results in weakness of the abductor mechanism of the affected hip. Total hip arthroplasty has been described as the ideal procedure in the management of these fractures and has been remains the gold standard in its treatment^6^. The other option to be considered in the management of these fractures would be to perform an osteotomy at the fracture site to undo the malunion and proceed to fix it with either surface or intramedullary implants but it would require more extensive dissection and would be associated with extensive bleeding as well as longer surgical time. Other procedures for the management of malunited fractures have not been described in a review of literature. In this scenario management of malunited intertrochanteric fractures posts a challenge regarding treatment options. Even though these patients don’t have significant pain, they have issues such as shortening of the affected limb, trendelenbergs gait due to proximal migration of the greater trochanter and abductor weakness and mechanical factors such as decrease of range of motion of the affected hip resulting in difficulty or inability to squat or sit cross legged which is disabling to the patient. All 15 patients in our study belonged to a rural population who are primarily involved in agricultural work and often depend on daily wages to support themselves.

The ability to squat and sit cross legged is essential for them to carry out their activities of work as well as daily living to the best possible extent. Hence total hip arthroplasty would not be an ideal treatment option for them due to factors such as lack of affordability and avoidance of squatting and sitting cross legged following the procedure which is an essential requisite for the study population. Valgus osteotomy is an established procedure which is usually performed in femoral neck non-unions and has not traditionally described for malunited intertrochanteric fractures. This osteotomy is performed at the subtrochanteric level and no effort is made to open the fracture site to undo the malunion. Valgus osteotomy has the advantages of correcting the limb length discrepancy, restores the reduced neck shaft angle to near normal, improves the range of movement and gives good functional results bringing about an improvement in the quality of the life for the patient enabling them to carry out their activities of work as well as daily living to the best possible extent without any restriction. In our procedure, we did not take a wedge of bone as described in the classical form of the procedure but instead we performed a sliding osteotomy in order to maintain length and to avoid shortening. We emphasise the fact that a sliding osteotomy is superior to a wedge osteotomy as it helps in maintain limb length to the best possible extent and also the fixation with a DHS is quite easy and straightforward as compared to an angle blade plate which is technically demanding and has quite a steep learning curve^7-10^. Fixation following osteotomy can be done with either a DHS or an angled blade plate. We routinely used a DHS with a fixed 135° angle as we found it to be technically easier to perform as compared to a blade plate which is more technically demanding.

In our study, there was a good restoration of the neck shaft angle to a near normal level in all patients and also good correction of the limb length discrepancy was achieved. Mild shortening was seen in three of our patients but it was quite well tolerated by them without any issues. There was a good improvement in the gait pattern in all patients following union of the osteotomy site and starting of full weight bearing walking which was at an average of 12.7 weeks. All patients had a significant improvement in the range of movement of the affected hip in the post-operative period (P<0.005) and at the end of three months they were able to squat and sit cross legged quite comfortably which was quite satisfactory for them. The increase in the range of motion of the affected hip is due to the better arc of movement and also due to the correction of the mechanical block of abduction and internal rotation caused by the coxa vara resulting from the malunion of these fractures. There was also an improvement in the Harris hip score with a gain of 18.67 points in the post-operative period. There were no iatrogenic complications seen in our study and none of our patients were lost to follow-up. All patients were followed-up for a three years period and were doing well functionally at the end of the last follow-up. A review of literature showed that valgus osteotomy with DHS fixation was performed mostly for non-unions of the femoral neck with adequate literature supporting the same^11-13^.

The limitations of our study were a small study sample with a relatively short follow-up period. A larger study group would ideally be essential to observe whether the beneficial effects of the osteotomy are uniform and consistent. Through this study, we conclude that Valgus osteotomy with DHS fixation is an effective treatment option in the management of malunited intertrochanteric fractures of the femur. It corrects the limb length discrepancy, restores the neck shaft angle and improves the range of motion thereby improves the function of the affected hip and improves the quality of life for the patient. It can be used as a good and reliable alternative in patients for whom Total hip arthroplasty would not be indicated.

## Conclusion

Valgus osteotomy with DHS fixation is an effective procedure in the management of malunited intertrochanteric fractures. It corrects the limb length discrepancy, restores the decreased neck shaft angle, improves range of movement, restores the integrity of the abductor mechanism of the hip and gives good functional results.
